# Image classification model based on large kernel attention mechanism and relative position self-attention mechanism

**DOI:** 10.7717/peerj-cs.1344

**Published:** 2023-04-21

**Authors:** Siqi Liu, Jiangshu Wei, Gang Liu, Bei Zhou

**Affiliations:** College of Information Engineering, Sichuan Agricultural University, Ya’an, Sichuan, China

**Keywords:** Attention mechanism, Image classification, Deep learning, Computer vision, Convolutional neural networks

## Abstract

The Transformer has achieved great success in many computer vision tasks. With the in-depth exploration of it, researchers have found that Transformers can better obtain long-range features than convolutional neural networks (CNN). However, there will be a deterioration of local feature details when the Transformer extracts local features. Although CNN is adept at capturing the local feature details, it cannot easily obtain the global representation of features. In order to solve the above problems effectively, this paper proposes a hybrid model consisting of CNN and Transformer inspired by Visual Attention Net (VAN) and CoAtNet. This model optimizes its shortcomings in the difficulty of capturing the global representation of features by introducing Large Kernel Attention (LKA) in CNN while using the Transformer blocks with relative position self-attention variant to alleviate the problem of detail deterioration in local features of the Transformer. Our model effectively combines the advantages of the above two structures to obtain the details of local features more accurately and capture the relationship between features far apart more efficiently on a large receptive field. Our experiments show that in the image classification task without additional training data, the proposed model in this paper can achieve excellent results on the cifar10 dataset, the cifar100 dataset, and the birds400 dataset (a public dataset on the Kaggle platform) with fewer model parameters. Among them, SE_LKACAT achieved a Top-1 accuracy of 98.01% on the cifar10 dataset with only 7.5M parameters.

## Introduction

Since the breakthrough made by AlexNet (Krizhevsky, Sutskever & Hinton, 2017), models based on convolutional neural networks (CNN) have gradually become the mainstay in computer vision tasks. It has shown advanced performance in tasks such as image classification (Hassan et al., 2022), object detection (Girshick, 2015), and instance segmentation (Nguyen et al., 2021). However, good performance is often accompanied by many model parameters and calculated quantities. Guo et al. (2022b) proposed a Visual Attention Network (VAN). VAN surpasses the current widely-known CNN-based and Transformer-based backbone networks on the ImageNet dataset (Deng et al., 2009) by using a Large Kernel Attention (LKA) module with very few model parameters and calculated quantity. In addition, with the advent of Vision Transformer (VIT) (Dosovitskiy et al., 2020), VIT attained excellent results compared to famous convolutional neural network models (He et al., 2016).

The above achievements have attracted significant attention among a large number of researchers. With further study of VIT, researchers found that VIT achieves excellent results in classification tasks. However, Yuan et al. (2021) reveals problems such as high model complexity, large computing resource consumption, and high training cost. In order to address these problems, researchers ameliorated the Self Attention module of the Transformer. They proposed a model (Liu et al., 2021a; Liu et al., 2021b) that achieves better results with fewer computing resources than VIT. Nevertheless, the model (Zhai et al., 2022) that only applies the VIT structure still has the disadvantages of high model complexity and high computational resource consumption. In order to further reduce the computational resources while retaining the model’s excellent performance, Srinivas et al. (2021) tried to introduce a Transformer structure from the CNN model. They used multi-head self-attention (MHSA) as a substitute for the convolution operation with the convolution kernel size of 3x3 in ResNet Bottleneck while making other parts of the network consistent with ResNet to form a new model. This model is called Bottleneck Transformer. It achieves 84.7% accuracy in ImageNet classification tasks, and its inference speed is 1.64 times faster than EfficientNet (Tan & Le, 2019). Meanwhile, Yuan et al. (2021) also proposed the CeiT model by introducing CNN from the Transformer model, and they changed the way of patch-to-token in VIT. Specifically, CeiT split the picture into patches after passing through the convolution layer and maximum pooling layer. Therefore, CeiT achieves excellent classification results at a smaller training cost. In addition, researchers found that CNN is good at capturing the details of local features but lacks the ability to obtain the global representation of features. On the contrary, although VIT has an excellent global representation of features, it is difficult to obtain the details of local features (Peng et al., 2021). Based on the characteristics of VIT, the previous work (Pan et al., 2022) has tried to introduce self-attention into CNN to capture the relationship between pixels that are far apart in the image better. Some work has also been done to apply CNN to Transformer to improve the model’s generalization ability and enhance its ability to extract local features (Guo et al., 2022a).

Inspired by the above works, we introduce the Transformer module with relative position self-attention variant used by CoAtNet_0 (Dai et al., 2021) to the Van_Tiny to build a model named Large Kernel Attention Convolution And Transformer (LKACAT), which combines both advantages of CNN and Transformer. Without additional training data, LKACAT achieves competitive results on the cifar10 dataset (Krizhevsky & Hinton, 2009), cifar100 dataset, and birds400 dataset (Moreno, 2022; Piosenka, 2023) with relatively small model parameters. In this research, we put forward a new model. The superiority of the CNN-Transformer hybrid model is verified on medium-sized datasets by us, and we provide a reference for other researchers.

## Background

This section presents a literature survey of some models that incorporate the advantages of CNN and Transformer. To enable the model to obtain local feature representation and feature global representation better, Li et al. (2022) tried to improve the model structure to fuse the advantages of CNN and Transformer and proposed a novel Unified Transformer (UniFormer). It integrates the advantages of 3D convolution and self-attention in the form of a Transformer and achieves more competitive accuracy in image classification tasks. However, UniFormer has not been greatly optimized in terms of the number of parameters. The Uniformer introduced the relation aggregator, which unified convolution and self-attention into token correlation learning. By designing local and global token affinity at both shallow and deep levels, UniFormer is able to achieve more efficient learning and presentation capabilities. In order to reduce the computational resource consumption while maintaining good recognition accuracy, Liu et al. (2021a) constructed Swin TransFormer, which achieves higher Top-1 accuracy than VIT by proposing a window-based attention computing approach while significantly reducing the computational resources required by the model. Although Swin Transformer achieves better results than VIT with smaller computational resource consumption, it still suffers from high time complexity and high space complexity compared with classical networks with CNN backbone such as EfficientNet.

Therefore, researchers tried introducing the self-attention mechanism in Transformer into the CNN backbone network to optimize the model. Liu et al. (2021b) attempted to incorporate the self-attentive mechanism in Transformer into CNN backbone networks by improving the model structure. They constructed a new hierarchical cascaded multi-headed self-attention module, which significantly reduces the time complexity and space complexity by hierarchical cascading. Furthermore, this module can be easily inserted into any CNN architecture. Based on this module, Liu et al. (2021b) proposed a backbone network called TransCNN (Liu et al., 2021a; Liu et al., 2021b), which perfectly inherits the advantages of CNN and Transformer. TransCNN substantially reduces the model’s time complexity and space complexity while maintaining high accuracy in classification tasks. Pan et al. (2022) tried to use convolutional operations to replace the self-attention mechanism by analyzing the operation process of the self-attention mechanism in a Transformer and achieved a high accuracy rate while significantly reducing the number of model parameters. In addition, Peng et al. (2021) build a parallel network structure that effectively combines convolutional operation and self-attention mechanism to enhance the model’s learning ability and representation ability of the model. This hybrid network structure is named Conformer, and it relies on Feature Coupling Units to fuse local feature representation and global feature representation in an interactive manner at different resolutions. On the ImageNet dataset, Conformer is 2.3% higher in Top-1 accuracy than DeiT with similar parameters and complexity.

As mentioned above, while many previous works have focused on model structure improvement and model fusion, our study focuses more on the attention mechanism. We improve and fuse the large kernel attention and self-attention mechanisms to achieve excellent recognition accuracy with a small number of parameters in our model.

## Methods

### Data preprocessing

To demonstrate the effectiveness of LKACAT, we separately use the cifar10 dataset, cifar100 dataset, and birds400 dataset, which vary widely from one another in the number of categories. The cifar10 dataset contains 10 different categories of objects. In comparison, the cifar100 dataset contains 100 different categories of objects, all composed of 60,000 colorful images with a resolution of 32 × 32 and a total of 50,000 training set images and 10,000 validation set images in each dataset. The birds400 dataset contains 400 species of birds with 58,388 training images, 2,000 testing images, and 2,000 validation images, all colorful images with a resolution of 224 × 224. For the birds400 dataset, we randomly redivided the entire dataset in the ratio of 8:2 into a training set and a test set. To enhance the model’s generalization ability and prevent over-fitting in the training process, we used random resize scale, color-jitter, random clipping, random horizontal flipping, label-smoothing (Müller, Kornblith & Hinton, 2019), mixup (Zhang et al., 2017), cutmix (Yun et al., 2019) and random erasing (Zhong et al., 2020) to enrich the training data. Our training scheme mostly follows DeiT (Touvron et al., 2021). Since the image size of the dataset we use is different from that of DeiT, we use the bilinear interpolation method to extend the resolution of all the images to 224 × 224 and change the random resize scale range of the images from [0.08,1.00] to [0.75,1.00] to fit our work.

### Model designing

In this part, we mainly discuss how to combine the CNN structure and Transformer structure better to design a new model. The superiority of CNN mainly depends on its two inherent inductive biases, translation invariance and local correlation. Van_Tiny makes full use of the advantages brought by CNN. Its LKA module decomposes the convolution operation into three parts, namely Depthwise Convolution (Chen et al., 2014), Depthwise and Dilated Convolution (Yu & Koltun, 2015), and Pointwise Convolution (Szegedy et al., 2015). Thanks to this decomposition method, Van_Tiny is more convenient for decomposing the convolution of a large kernel (Ding et al., 2022). Therefore, Van_Tiny maintains the advantages of CNN while expanding its receptive field. In addition, the LKA module introduces spatial attention and channel attention to capture the relationship between pixels at different positions in the feature map, enhancing the feature extraction ability of the model. The formula of depthwise convolution is shown in Eq. (1):

(1) \begin{eqnarray*}{y}_{i}=\sum _{j\in l \left( i \right) }{w}_{i-j}\odot {x}_{j},\end{eqnarray*}

where *x*_*i*_, *y*_*i*_ are the input and output at position *i*, respectively, *w*_*i*−*j*_ is the weight matrix at position *i* − *j*, and *l* (*i*) represents the local neighborhood of *i*.

Although Van_Tiny has a larger receptive field, it is still inadequate compared to the global receptive field of Transformer. In addition, Transformer’s self-attention weight dynamically depends on the input. Therefore, Transformer captures the complex relationship between different spatial locations more easily than CNN. The formula of the self-attention mechanism is shown in Eq. (2):

(2) \begin{eqnarray*}{y}_{i}=\sum _{j\epsilon g} \frac{\mathit{exp} \left( {x}_{i}^{T}{x}_{j} \right) }{\sum _{k\epsilon g}\mathit{exp} \left( {x}_{i}^{T}{x}_{k} \right) _{{A}_{i,j}}} {x}_{j},\end{eqnarray*}

where *g* represents the global space, *A*_*i*,*j*_ represents the attention weight, (*x*_*i*_, *x*_*j*_) denotes an arbitrary position pair, and *x*_*i*_, *y*_*i*_ are the input and output at position *i*, respectively.

CoAtNet_0 used the Transformer blocks equipped with relative position self-attention variant, in which the global static convolution kernel and adaptive attention matrix are added so that the attention weight is jointly decided by the *w*_*i*−*j*_, which guarantees the translation invariance in Eq. (1), and the ${x}_{i}^{T}{x}_{j}$, which provides adaptive input weight in Eq. (2). Therefore, this Transformer block with relative position self-attention variant has better generalization ability, which makes up for the deficiency of the Transformer to some degree.

We decided to use the above two structures to build the backbone network of the new model. In short, we need to consider two questions: 1. How to effectively combine the LKA module with the Transformer block with a relative position self-attention variant? 2. How to achieve higher classification accuracy on the new model with fewer parameters?

### Decomposing convolution kernel to control model size

In order to control the size of the model, we decided to use the Van_Tiny network with a small number of parameters as the basic framework of the new model. The Van_Tiny model consists of four primary stages, each of which will reduce the spatial resolution of the feature map and increase the number of channels. In each LKA module in Stage, we sequentially connect Depthwise Convolution with a convolution kernel size of 5 × 5, Depthwise and Dilated Convolution, which convolution kernel size of 7 × 7 and dilation degree of 3 and a Pointwise Convolution to supersede the convolution operation with a convolution kernel size of 21 × 21.

To facilitate the calculation of the parameters, we omitted the bias in reasoning about the parameters and assumed that the input and output features have the same size (CxHxW). The convolution parameters decomposed into three parts in LKA can be calculated by Eq. (3):

(3) \begin{eqnarray*}Params= \frac{K}{d} \times \frac{K}{d} \times C+(2d-1)\times (2d-1)\times C+C\times C\end{eqnarray*}

where d represents the expansion rate, K represents the convolution kernel size, and C represents the number of image channels.

Under the above conditions, Eq. (4) for calculating the parameters of ordinary convolution is:

(4) \begin{eqnarray*}Params=K\times K\times C\times C\end{eqnarray*}

where k represents the convolution kernel size, and c represents the number of image channels.

It can be deduced from Eqs. (3) and (4) that when *K* = 21 and *d* = 3, the large kernel convolution decomposed by the LKA module reduces the model’s parameters while retaining the original receptive field.

### Using the Transformer variant to improve classification accuracy

**Figure 1:**
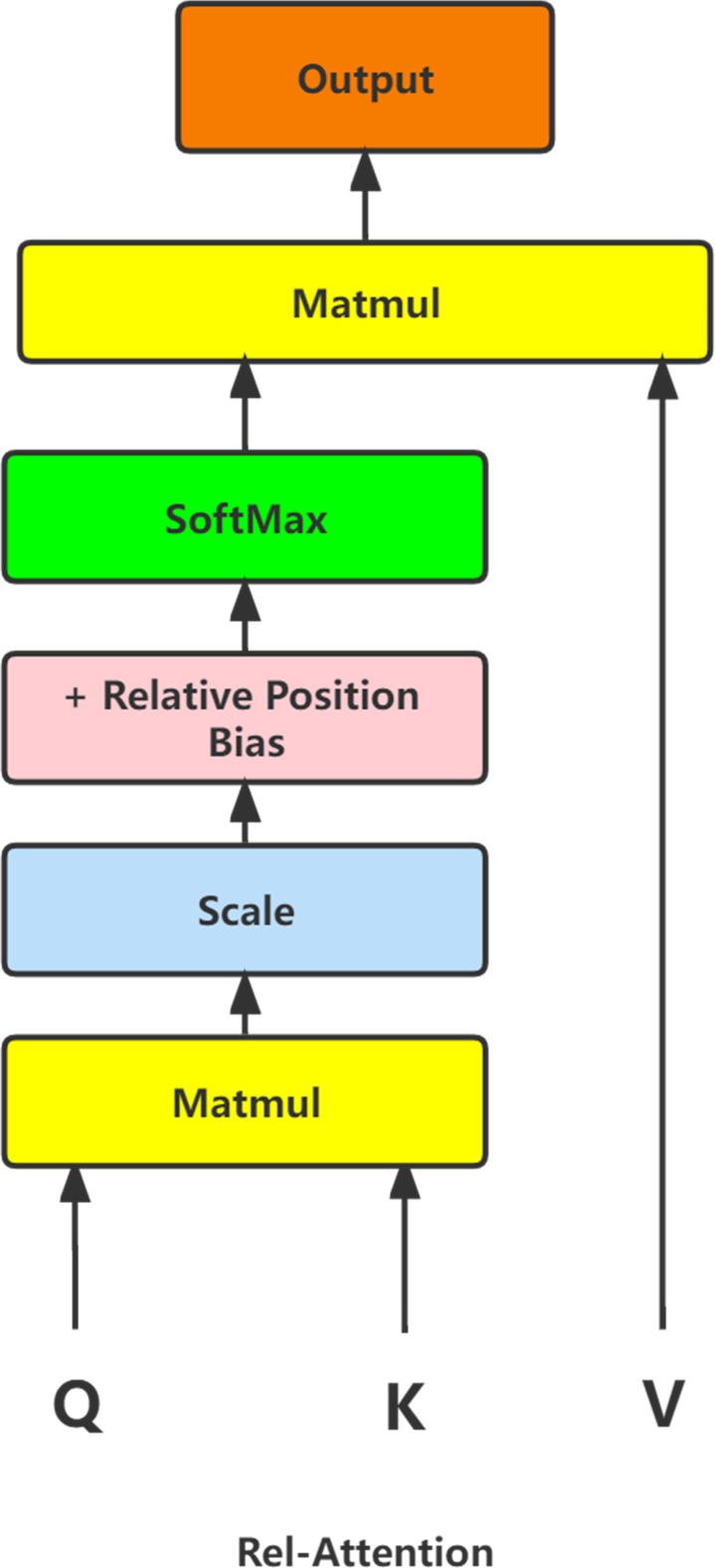
The structure diagram of Rel-Attention.

We try to improve the model classification accuracy by introducing a Transformer block with a relative position self-attention variant in the structure of Van_Tiny. As shown in Fig. 1, this Transformer block uses relative position bias during the self-attention phase. When the width and height of the input image are respectively W and H, define a weight matrix with the size of [2*H-1,2* W-1], then regard the input image as the convolution kernel in the convolution operation and the weight matrix as the convoluted image. The convolution operation is completed when the stride is equal to 1. Each convolution operation obtains the output feature map with the same size as the input feature map. Finally, the results of each convolution operation are spliced to generate a relative weight matrix with the size of [W*H,W*H]. Therefore, the weights of the point-to-point mapping in the feature map can be found in the relative weight matrix. In order to introduce the relative weight matrix into the self-attention mechanism of the Transformer more naturally, we added the relative position bias obtained by matrix transformation of the relative weight matrix to the multiplication result of the scaled Q matrix and K^T^ matrix. We put this result into the SoftMax function and multiply it with the V matrix to get the self-attentive matrix with CNN advantage.

 Transformer block with relative position self-attention variant has better generalization ability, further improving model classification accuracy.

### Module fusion and improvement

**Figure 2:**
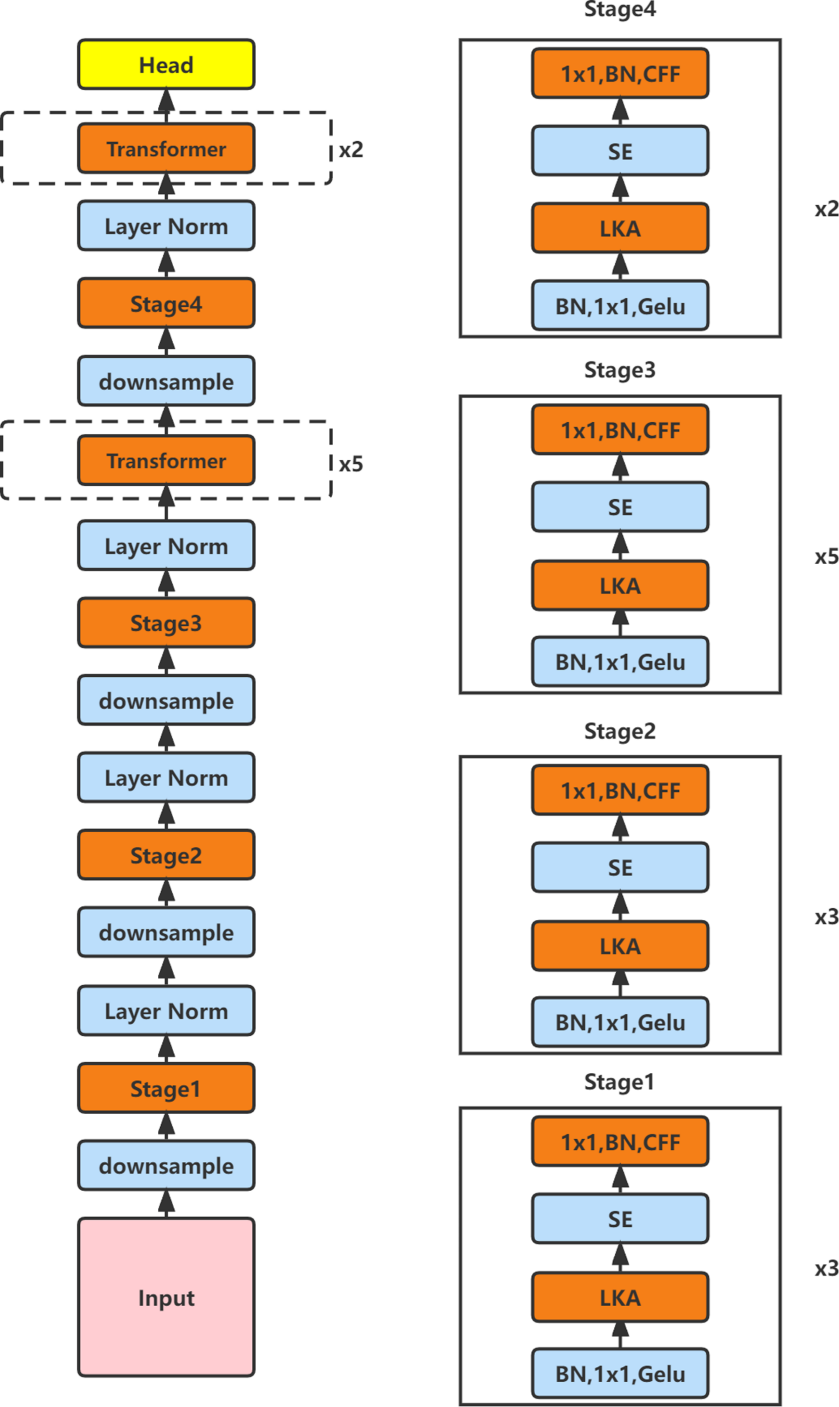
The structure diagram of SE_LKACAT.

In order to make the new model possess the strengths of CNN and Transformer while maintaining a small number of parameters, we consider inserting Transformer blocks with relative position self-attention variants into the Van_Tiny model. As shown in Fig. 2, we constructed a model named SE_LKACAT, which was inspired by ConvNext (Liu et al., 2022) to vertically stack the transformer blocks and convolutional layer with the LKA module in a ratio of 3:3:10:4. Since the feature map size is constant through the Transformer block with the relative position self-attention variant, we try to insert the Transformer block after the convolution layer when the size of the feature map reaches a controllable level by the convolution operation. When the feature map size is 14 × 14, we use five vertically stacked Transformer blocks as the inserted calculation blocks. In addition, when the feature map size is 7 × 7, we insert two vertically stacked Transformer blocks as the calculation blocks. In this way, by introducing the Transformer block into the Van_Tiny model, the strengths of CNN and Transformer are combined to improve the classification ability of the model.

The model maintains a large receptive field and obtains spatial and channel attention via the LKA module in the convolution stage. However, considering the overall structure of the model, we believe that the model needs more channel attention in the convolution stage to improve the feature representation ability. To determine the adding location of the channel attention, after a series of experiments, we found that adding the channel attention module after the LKA module achieved the best results. We try to send each feature map that passes the LKA module into the Squeeze-and-Exclusion (SE) module (Hu, Shen & Sun, 2018) to filter out nonsignificant channel information to prepare for the calculation of the feature map in the Transformer block. In addition, the model constructed by us without the SE module is named LKACAT.

The SE module first performs a global pooling operation on the feature map (the size is C*H*W) to obtain a feature map with the size of 1*1*C and then sends the feature map to two fully connected layers, the number of neurons in the first fully connected layer is $ \frac{c}{16} $. The number of neurons in the second layer is reset to C. The complex correlation between channels can be better fitted by adding more nonlinear processing through two fully connected layers. Finally, the feature map is put into a Sigmoid layer to get a feature map with a size of 1*1*C, and then the feature map and the original image are matrices multiplied to get the channel attention of the feature map.

We introduce the SE module into the convolution stage of LKACAT to enhance the channel’s attention to better capture the details of the local features of the feature map and the optimized model named SE_LKACAT.

## Result

### Model evaluation index

We evaluate the model using three commonly used classification task evaluation indicators, namely Top-1 accuracy, Top-5 accuracy, and the number of parameters. Among them, the number of parameters refers to the total number of parameters that need to be trained in the network model, which is related to the space complexity of the model operation. It visually shows the size of the network model. And accuracy is the most common classification evaluation index, and it is shown in Eq. (5):

(5) \begin{eqnarray*}Accuracy= \frac{TP+TN}{P+N} \end{eqnarray*}

where TP is the positive samples predicted by the model as the positive class, TN is the negative samples predicted by the model as the negative class, P is all positive samples, and N is all negative samples.

In simple terms, accuracy is the number of samples correctly classified by the model divided by the number of all samples. Top-5 accuracy refers to that among the test picture’s N (N>5) classification probabilities, and the top five largest classification probabilities are taken to check whether the correct label is included in them. If so, the prediction is correct; otherwise, the prediction is wrong. It is shown in Eq. (6):

(6) \begin{eqnarray*}Top5~Accuracy= \frac{Fiv{e}^{(TP+TN)}}{P+N} \end{eqnarray*}

where *Five*^()^ represents the number of correct labels included in the top five categories with the largest classification probability.

Like Top-5 accuracy, Top-1 accuracy refers to taking the largest classification probability among N (N>1) classification probabilities of the test picture to check whether its category matches the correct label. If so, the prediction is correct; otherwise, the prediction is wrong. It is shown in Eq. (7):

(7) \begin{eqnarray*}Top1~Accuracy= \frac{Firs{t}^{(TP+TN)}}{P+N} \end{eqnarray*}

where *First*^()^ represents the number of labels matching the maximum classification probability.

The above three evaluation indexes show high reference values in the field of the classification task. Among them, the higher the percentage score of Top-1 accuracy and Top-5 accuracy, the stronger the classification ability of the model, while the smaller the number of parameters, the lower the space complexity of the model.

### Analysis of experimental results

The experimental results are shown and analyzed in this part to prove the validity of the LKACAT model. We conducted experiments on three datasets: cifar10, cifar100, and birds400. In order to ensure the fairness of the experiment, all models in our experiments used the same training method to complete the training, and we did not use the pre-training method for the experiment. In addition, we take the highest accuracy in the cooldown-epochs as the final accuracy.

We observed the training process of Van_Tiny, CoAtNet_0, and LKACAT on the cifar100 dataset, cifar10 dataset, and Birds400 dataset. We found that the test_loss and train_loss maintained the same decreasing trend and fit together well for all training processes of these three models. In addition, when the training proceeded to the interval of 250 epochs—300 epochs, the accuracy of the models was basically stable, while the test_loss and train_loss kept unchanged basically.

Based on the above observations, we believe that after 310 epochs of training, the train_loss and test_loss of the three models gradually decreased and fit well. At the same time, the Top-1 accuracy gradually tends to be constant. This phenomenon indicates that the above three models converge well.

In Table 1, we compare the performances of LKACAT, Van_Tiny, and CoAtNet_0 on three datasets, and it can be clearly observed that LKACAT achieves higher Top-1 accuracy than Van_Tiny. On the cifar100 dataset, the Top-1 accuracy of LKACAT is 2.34% higher than that of Van_Tiny. However, the Top-1 Accuracy of LKACAT on the birds400 dataset is 0.05% lower than the Top-1 Accuracy of CoAtNet_0, and it achieves better results on the cifar10 and cifar100 datasets. In addition, as shown in Table 2, when the number of parameters of LKACAT is less than half of that of CoAtNet_0, LKACAT has achieved Top-1 Accuracy, which is better than Van_Tiny and similar to CoAtNet_0.

**Table 1:** Performances of LKACAT, Van_Tiny and CoAtNet on cifar10, cifar100 and Birds400.

DataSet	Method	Top-1 Accuracy	Top-5 Accuracy
cifar10	Van_Tiny	97.67%	99.88%
	CoAtNet	97.67%	99.91%
	LKACAT	**97.89%**	**99.94%**
cifar100	Van_Tiny	82.99%	96.39%
	CoAtNet	85.03%	**96.69%**
	LKACAT	**85.33%**	96.37%
Birds400	Van_Tiny	97.17%	**99.44%**
	CoAtNet	**97.41%**	**99.44%**
	LKACAT	97.36%	99.43%

**Notes.**

The best results are in bold.

**Table 2:** Comparison of parameters between LKACAT and classical models on Birds400.

Method	Van_Tiny	CoAtNet	LKACAT	SE_LKACAT	ResNext50	ResNet50	SE_ResNext50	SE_ResNet50
Parameters(M)	3.9	17.3	7.5	7.5	23	24	28	29

**Notes.**

The numbers in the table represent the total parameters.

In order to observe the benefits of using SE channel attention to the LKACAT model, we list the different performances of SE_LKACAT and LKACAT in the three datasets in Table 3. In addition, we compare the proposed model with some classical models, and it is evident that our model achieves higher classification accuracy with a smaller number of parameters.

**Table 3:** Performances of SE_LKACAT, LKACAT and some classical models on cifar10, cifar100 and Birds400 datasets.

DataSet	Method	Top-1 Accuracy	Top-5 Accuracy
cifar10	LKACAT	97.89%	**99.94%**
	SE_LKACAT	**98.01%**	**99.94%**
	SE_ResNext50	97.20%	99.85%
	SE_ResNet50	95.75%	99.87%
	ResNext50	96.88%	99.76%
	ResNet50	96.62%	99.73%
cifar100	LKACAT	85.33%	**96.37%**
	SE_LKACAT	**85.39%**	96.35%
	SE_ResNext50	83.35%	96.19%
	SE_ResNet50	82.78%	96.22%
	ResNext50	81.40%	94.88%
	ResNet50	80.76%	92.12%
Birds400	LKACAT	**97.36%**	99.43%
	SE_LKACAT	97.22%	99.30%
	SE_ResNext50	97.29%	**99.47%**
	SE_ResNet50	96.98%	99.34%
	ResNext50	97.33%	99.39%
	ResNet50	97.14%	99.37%

**Notes.**

The best results are in bold.

## Discussion

The results obtained by using LKACAT to complete the classification task on medium-sized datasets are shown in Tables 1 and 3. It can be observed that LKACAT is not only superior to VAN_Tiny and CoAtNet_0, which provide inspiration for its construction but also achieves better results on some classical models. In this research, we reduced the model deviation and improved the classification performance by integrating existing excellent models and further achieved better classification results by combining the attention module. The integration model based on Van_Tiny and CoAtNet_0 proposed in this paper is superior to some classical, traditional methods and achieves better results after combining attention modules. Although this model performed well, there are still some shortcomings. For instance, LKACAT is ineffective on large datasets such as ImageNet compared with some classical models.

In addition, the LKACAT model has not been pre-trained, which may affect the model’s accuracy. Therefore, we will use the pre-training method to improve the efficiency and stability of LKACAT in the following research stage.

## Conclusions

In this work, we try to integrate the long-distance feature dependence brought by the relative position self-attention mechanism into the convolutional network under a large receptive field, making our model inherit both advantages. We regularly introduce Transformer blocks with relative position self-attention variants into the Van_Tiny network to build a new model. Without additional training data, this model achieves better Top-1 accuracy than Van_Tiny and CoAtNet_0 on cifar10 and cifar100 with a smaller number of parameters. Our experiments show that our model effectively combines the advantages of CNN and Transformer, and we verify the superiority of this model over other classical models in the meantime.

In future work, we will build more lightweight, excellent models by increasing the stacking ratio of modules. In addition, since our model does not perform well in classification tasks on large datasets, residual networks can improve accuracy on large datasets by increasing the depth. Therefore, we would like to introduce residual networks to increase network branches, thus forming a multi-branch network that fuses feature information learned from different branches (Lin et al., 2022) to complete classification tasks on large datasets better.

## Additional Information and Declarations

### Data Availability

The following information was supplied regarding data availability: The resplitted_birds400 dataset is available at Zenodo: Liusiqi. (2022). The repartitioned birds400 dataset [Data set]. Zenodo. https://doi.org/10.5281/zenodo.7197318
The birds400 dataset is available at Kaggle: https://www.kaggle.com/datasets/antoniozarauzmoreno/birds400
The cifar10 and cifar100 datasets (labeled subsets of the 80 million tiny images dataset collected by Alex Krizhevsky, Vinod Nair, and Geoffrey Hinton) are available at the University of Toronto:
http://www.cs.toronto.edu/ kriz/cifar.html
LKACAT code is available at Zenodo: Liusiqi. (2022). LKACAT. Zenodo. https://doi.org/10.5281/zenodo.7227119
